# Noisy anthropogenic infrastructure interferes with alarm responses in Savannah sparrows (*Passerculus sandwichensis*)

**DOI:** 10.1098/rsos.172168

**Published:** 2018-05-16

**Authors:** Bridget Antze, Nicola Koper

**Affiliations:** Natural Resources Institute, University of Manitoba, 303-70 Dysart Road, Winnipeg, Manitoba, Canada R3T2N2

**Keywords:** anthropogenic noise, anti-predator behaviour, distracted prey hypothesis, acoustic masking, natural gas compressor stations, petroleum

## Abstract

Many birds rely on anti-predator communication to protect their nests; however, anthropogenic noise from industrial activities such as oil and gas development may disrupt acoustic communication. Here, we conducted acoustic playback experiments to determine whether Savannah sparrows (*Passerculus sandwichensis*) responded to conspecific alarm calls by delaying feeding visits, and whether this response was impaired by noise-producing natural gas compressor stations, generator- or grid-powered screw pump oil wells, and noise amplitude. We played alarm calls, and, as a control, western meadowlark songs, to Savannah sparrows as they approached their nests to feed their nestlings, and measured feeding latency. The greatest impacts on behaviour were detected at the noisiest treatment, compressor stations; feeding latency was shortened here compared with control sites, which may expose nests to greater predation risk. As noise amplitudes increased, Savannah sparrows took longer to feed following meadowlark playbacks, perhaps because noise interfered with interpretation of acoustic cues. The effects of compressor stations on anti-predator behaviour may be best explained by the distracting effects of anthropogenic noise, while increases in feeding latency following meadowlark playbacks may be explained by a heightened response threshold caused by acoustic masking. Industrial infrastructure can influence the reproductive success of wildlife through its impact on perception and interpretation of conspecific signals, but these effects are complex.

## 1. Background

Many animals rely on alarm calls to deter predators [1], solicit help from conspecifics [2], and to warn neighbours [3], mates [4] and offspring [5] about impending dangers. Alarm calls may encode complex information regarding urgency [6], size [2], type of predator [7] or predator behaviour [8]. Many species respond differently to different types of alarm calls [2,7], hence communicating anti-predator information accurately is necessary to elicit the appropriate response from conspecifics. Responding to a potential predator can have life or death consequences, so animals are under selective pressure to ensure they are able to detect and react quickly to alarm calls [9]. Indeed, higher rates of alarm calling during nest defence have been linked to improved nest survival for both red-winged blackbirds (*Agelaius phoeniceus*) [10] and American goldfinches (*Spinus tristis*) [11], suggesting that, for many bird species, effective anti-predator communication is an important determinant of reproductive success. However, several factors may impair the ability of animals to display appropriate anti-predator behaviour, including hunger [12], distracting stimuli [13] and the presence of ambient anthropogenic noise [14].

As increasing urbanization and industrial development encroach on natural areas, there is growing concern that anthropogenic noise may interfere with acoustic communication [15,16]. Impaired communication resulting from anthropogenic noise has been linked to lower lek attendance in greater sage-grouse (*Centrocercus urophasianus*) [17], reduced pairing success in ovenbirds (*Seiurus aurocapilla*) [18] and impaired nestling development in house sparrows (*Passer domesticus*) [19], indicating that the impacts of noise on communication have the potential to interfere with reproductive processes. The effects of noise on acoustic communication in individual species may lead to further impacts on ecological communities, such as declines in occupancy and abundance of boreal songbirds [20] and changes in avian community composition and species interactions [21]. However, to our knowledge, no previous studies have compared the effects of noise from different types of *in situ* industrial infrastructure on responses to alarm calls. The effects of noise are predicted to vary with amplitude, frequency and predictability of sound [22], and might even be influenced by the vertical structures that emit noise, which can cause reflectance and reverberation of sounds [23–26]. Therefore, different types of industrial infrastructure might be expected to have varying impacts on acoustic communication. Comparing among them will help identify mechanisms that explain ecological effects of anthropogenic noise, which in turn will help us identify effective mitigation measures.

One mechanism by which noise can interfere with acoustic communication is through frequency masking. Masking occurs when background noise overlaps with acoustic signals, thereby lowering the signal-to-noise ratio [25] and preventing animals from appropriately detecting or discriminating information within signals. Masking can have several consequences for communication. In extreme situations, masking may prevent animals from detecting signals. For example, nestling tree swallows (*Tachycineta bicolor*) failed to detect parents arriving at the nest to feed them [27], and failed to detect parental alarm cues [14] more frequently when exposed to elevated levels of ambient noise. When vocalizations are partially masked, noise may also prevent animals from discriminating more complex information encoded within signals [25]. For example, avian mate-attraction songs can include subtle information regarding male quality, which may be lost if songs are partially masked. Male ovenbirds that established territories close to natural gas compressor stations had lower mating success than those living in quieter areas, a finding that has been attributed to this phenomenon [18]. Finally, animals exposed to noise may decrease their response threshold, leading to a heightened rate of ‘false alarms' [28], whereby animals inappropriately respond to biologically irrelevant sounds when it is not adaptive to do so [12].

An alternative mechanism by which noise may prevent animals from responding appropriately to acoustic signals is through distraction. According to the ‘distracted prey hypothesis' [29] anthropogenic noise can provide an additional stimulus that animals must focus on, drawing attention away from other important tasks such as predator avoidance. Distraction acts differently from acoustic masking in that it does not prevent animals from actually perceiving acoustic signals, but it impairs their ability to focus attention on composing an appropriate response [30]. For example, Caribbean hermit crabs (*Coenobita clypeatus*) were less responsive to a silent looming object when exposed to playbacks of motor boat noise than they were during silence, even though noise did not hinder their ability to see the object [29]. Another important aspect of the distracted prey hypothesis is that, as long as noise can be perceived by an animal, it has the potential to distract them [29]. Therefore, the degree to which anthropogenic noise distracts may not be directly correlated with the amplitude of noise, as long as it can be heard [31]. Because the effects of distraction do not necessarily decline directly with declines in noise amplitude, a much greater extent of the landscape may be influenced by distraction than masking; as such, distraction may have greater conservation implications than does masking.

Savannah sparrows (*Passerculus sandwichensis*) are a common and widespread grassland songbird, breeding in open grassy habitats throughout Canada and the northern USA. Like other ground-nesting birds, Savannah sparrow nests are extremely vulnerable [32], so they must rely on camouflage and behavioural mechanisms for protection. When a predator is detected, Savannah sparrows emit a continuous stream of high-pitched chipping alarm calls until the danger has passed [32]. However, there are no studies that we are aware of that have experimentally tested how Savannah sparrows respond to alarm calls, or how alarm calls function in nest defence.

While numerous previous studies have demonstrated that bird species are capable of altering the structure of vocalizations in noise, the majority of work has focused on territorial and mate attraction songs [15,33–35], while the body of work examining the effects of noise on alarm calls is comparatively much smaller [14,36–38]. Furthermore, very few studies have directly tested whether anthropogenic noise prevents birds from responding appropriately to acoustic signals [5]. Establishing whether anthropogenic noise actually impairs communication is essential to determining the degree to which industrial noise may impact fitness. Understanding the effects of anthropogenic noise on anti-predator communication systems is particularly crucial, given the importance of responding appropriately to alarm calls to survival [10,11].

Here, we investigated whether industrial noise from petroleum (oil) wells and shallow natural gas (gas) compressor stations prevent Savannah sparrows from responding appropriately to conspecific alarm calls at nests. We first determined how Savannah sparrows respond to conspecific alarm calls under natural conditions. We broadcast recordings of conspecific alarm calls, and, as a control, the songs of a western meadowlark (*Sturnella neglecta*) (another common grassland bird found throughout the region), to Savannah sparrows prior to provisioning visits. We predicted that Savannah sparrows would respond to alarm calls by delaying feeding visits, to avoid drawing the attention of predators to their nests. To determine whether oil and gas infrastructure noise interfered with responses to alarm calls, we conducted the same experimental protocol on sites containing active, noise-producing infrastructure. We predicted that if infrastructure noise prevents Savannah sparrows from responding appropriately to alarm calls, Savannah sparrows would show less of a delay close to noisier infrastructure and in areas characterized by louder ambient noise.

## 2. Methods

### 2.1. Study area

**Figure 1.**
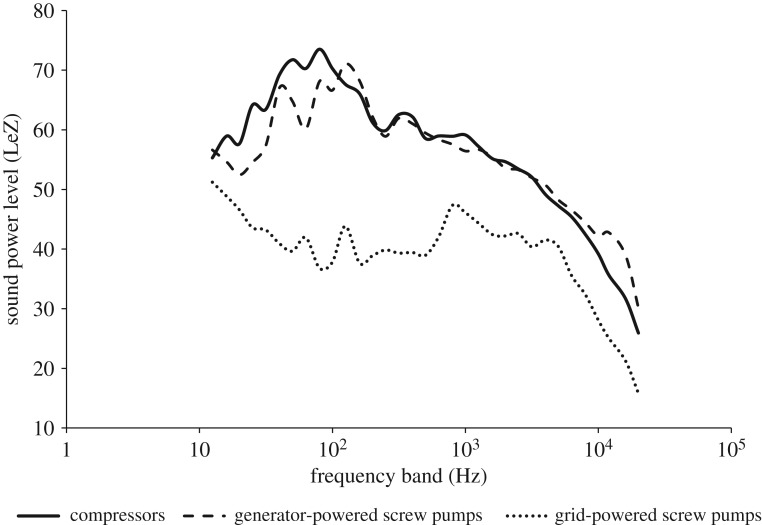
Sound profiles for ambient noise recorded 10 m from compressor stations (*n* = 4), grid-powered screw pumps (*n* = 5) and generator-powered screw pumps (*n* = 5). Measurements were made during April–August of 2013 and 2014 at representative infrastructure sites located within the mixed-grass prairie surrounding Brooks, Alberta, Canada (50.5642° N, 111.898° W). Measurements were taken at each one-third octave frequency band using a Bruel and Kjaer 2250 SPL meter and frequency analyser (dB(Z)), and averaged across all replicates for each infrastructure type.

The research took place in native mixed-grass prairies in a 200 km radius surrounding Brooks, Alberta, Canada (50.5642° N, 111.898° W), during May–July of 2013 and 2014. Increasing development from the oil and gas industry is prominent in this area, and oil and gas extraction structures such as screw pump oil wells and natural gas compressor stations are common throughout the region. Screw pumps are relatively small (1 m tall) oil extraction structures that rely on positive displacement to draw oil from the ground through one or more rotating screws [39]. The amplitude of noise produced by screw pumps is dependent on how they are powered. Screw pumps connected to the power grid (grid-powered screw pumps) are quieter (55 ± 2 s.e. dB(A) or 59 ± 1 s.e. dB(C) at 10 m; reference level: 20 µPa) than those powered by generators (generator-powered screw pumps, 68 ± 1 s.e. dB(A) or 79 ± 1 s.e. dB(C) at 10 m; reference level: 20 µPa). Compressor stations are taller and wider than screw pumps, often consisting of multiple structures including sheds and other buildings. Compressor stations pressurize natural gas through a combination of motors and turbines, and produce louder noise than screw pumps (69 ± 1 s.e. dB(A) or 82 ± 2 s.e. dB(C) at 10 m; reference level: 20 µPa). Energy is concentrated within the lower frequency ranges for all infrastructure treatments, with compressors producing their greatest amplitudes at the lowest frequencies, followed by generator, then grid-powered screw pumps (figure 1). The research took place on control sites, which were 200 × 800 m plots of native mixed-grass prairie located more than 800 m from all paved roads and noise-producing infrastructure (approx. 33 dB(A) or 52 dB(C), *n *= 12 sites), and infrastructure treatment sites, which were 200 × 800 m plots of native mixed-grass prairie centred around one of three infrastructure treatments: compressor stations (*n* = 4 sites), generator-powered screw pumps (*n* = 4 sites) and grid-powered screw pumps (*n* = 5 sites). On the control sites, ambient noise came from mostly natural sources such as wind, insects and birdsong, while ambient noise on oil and gas sites originated from infrastructure, in addition to natural noise sources.

### 2.2. Response to alarm calls under natural conditions

Before establishing whether noise interferes with alarm communication, it was necessary to establish how Savannah sparrows respond to alarm calls under natural conditions. Because Savannah sparrows build their nests directly on the ground, making them extremely vulnerable to predators, we predicted that Savannah sparrows would respond to alarm calls by delaying feeding visits, to avoid revealing their nest's location. To test this hypothesis, we conducted playback trials at 21 nests on control sites.

Our first step was to create playback recordings for the experimental trials. We created six alarm exemplars by recording alarm calls from Savannah sparrows in the same geographical region where experiments took place, but from different sites to ensure that playbacks were not of neighbours. To record alarm calls, we approached and stood within 1 m of active Savannah sparrow nests. When an adult approached within 10–15 m and commenced alarm calling, we used a Zoom H4N Handy Portable Digital Recorder with built-in microphone (Zoom Corporation, Tokyo, Japan), set in an XY stereo microphone configuration (90°) at the maximum recording volume, to record alarm calls, pointing the microphone directly at the bird. Digital recordings were saved as uncompressed WAV files at a sample rate of 48 kHz with 16-bit resolution. As a control, we recorded songs of four western meadowlarks (also in the region but not on study sites). We approached singing males as close as possible (20–30 m), and used the same equipment and procedures to record meadowlark songs as we used to record Savannah sparrow alarm calls. Recordings were uploaded into Raven Pro 1.5 for processing. We used a band filter to remove background noise 2 kHz above, 2 kHz below and between vocalizations. Alarm calls were amplified to ensure they could be played back at natural levels (approx. 80 dB(C) at 1 m).

**Figure 2.**
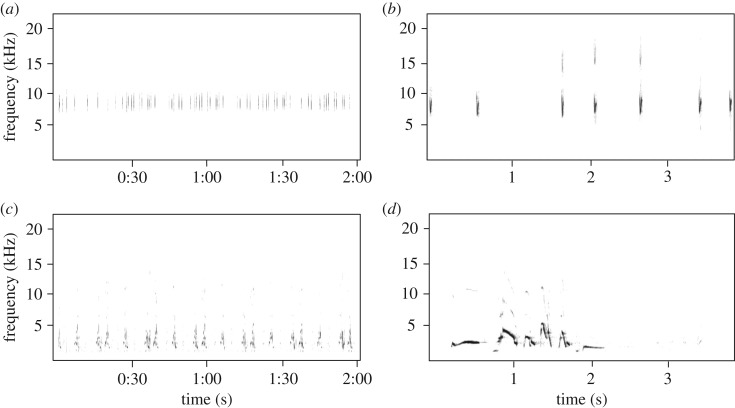
Spectrograms of playback recordings illustrating representative examples of (*a*) a full 2 min and (*b*) 3 s excerpt of a Savannah sparrow (*Passerculus sandwichensis*) alarm call playback, and (*c*) a full 2 min and (*d*) 3 s excerpt of a western meadowlark song playback. Recordings were made from field sites within the vicinity of Brooks, Alberta, Canada (50.5642° N, 111.898° W), during June 2013. The spectrograms were produced in Raven Pro 1.5 using a Hann window function with a fast Fourier transformation length of 512 samples, 3 dB bandwidth set at 135 Hz and overlap of 50%.

To control for changes in parental investment over the course of the nesting cycle, we conducted all playback experiments when nestlings were 5 days old. We placed a digital recorder (Zoom H4N Handy Portable Digital Recorder with a built-in microphone; Zoom Corporation, Tokyo, Japan) 30 cm from the nest, and a speaker (PureAcoustics HipBox Portable Audio Speaker, PureAcoustics Inc., Brooklyn, NY, USA) 2 m from the nest. The speaker was attached by an extension cable to an mp3 player (Apple iPod shuffle A1373: 2012–09; Apple Inc., Cupertino, CA, USA) containing one of the six possible alarm exemplars and, as a control, one of the four possible western meadowlark song bouts (figure 2). Playbacks were broadcast at approximately 80 dB(C) at 1 m, which is consistent with natural amplitudes for Savannah sparrow alarm calls that we recorded. We operated the iPod from a blind positioned 17–20 m from the nest. The digital recorder was also attached by an extension cable to a set of headphones, so we could hear when nestlings were begging, or when a parent arrived at the nest.

After a 10 min period to allow birds to acclimatize to the presence of the blind and equipment, we documented feeding behaviour on four provisioning visits, recording all movements within a 15 m radius of the nest as parents approached to feed. On the fifth visit, when a parent arrived at a perch carrying food within a 15 m radius of the nest, we broadcast the first playback recording for 2 min (either Savannah sparrow alarm calls or western meadowlark songs, alternated among nests), and measured the time it took the parent to travel to the nest to feed. We recorded behaviour on two more feeding visits, and then allowed a period of 5 min for the birds to return to baseline behaviour before repeating the same procedure with the second playback recording. We chose to use a playback duration of 2 min as pilot trials in June 2013 demonstrated that Savannah sparrows typically alarm-called for 2 min or longer when we approached their nests. The control playback duration of western meadowlark songs was also set at 2 min for consistency, and this is also within the normal range of song bout duration for this species. The time required to return to baseline behaviour (regular feeding intervals, no alarm calling) was also determined from pilot trials during June 2013.

We characterized responses of Savannah sparrows to stimuli by their feeding latency, the time (in seconds) it took sparrows to travel from a perch within a 15 m radius of the nest to the nest, following the onset of the conspecific alarm call or western meadowlark song playbacks. Individuals that took longer than 10 min (600 s) to feed following the onset of playback recordings were assigned a feeding latency value of 600 s (occurred at seven out of 60 nests, four following alarm playbacks, three following western meadowlark song playbacks). Feeding latency following each playback type was compared with feeding latency on baseline feeding visits, when no stimuli were played. A baseline feeding visit was the visit immediately prior to one on which a playback was broadcast, and baseline feeding latency was calculated as the time it took Savannah sparrows to travel to the nest from the same perch distance as on playback feeding visits. Observations from baseline feeding visits prior to both alarm and western meadowlark playbacks were combined, as there was no difference in the time it took Savannah sparrows to travel to the nest between pre-alarm playback and pre-western meadowlark song playback visits (*β *= 0.1859, s.e.* *= 0.2840, Wald's *χ*^2 ^= 0.43, *p *= 0.5129).

We dictated all observations into an Olympus VN-702PC digital voice recorder. We also recorded the date, time, site ID and nest ID.

### 2.3. Effects of oil and gas infrastructure on alarm responses

To determine whether infrastructure noise interferes with Savannah sparrow responses to alarm calls, meadowlark songs or behaviour on baseline feeding visits, we conducted the same experimental protocol as above, at an additional 38 nests at sites containing active, noise-producing infrastructure (*n*_compressor stations_ = 15 nests, *n*_generator‐powered screw pumps _= 11 nests, *n*_grid‐powered screw pumps_ = 12 nests). We recorded the distance and direction to infrastructure for each experimental nest, as well as the date, time, site ID and nest ID, as above.

### 2.4. Ambient noise

To quantify ambient noise levels during playback experiments, immediately following experimental trials we used a Zoom H4N digital recorder to record 30 s of ambient noise at a perch location within approximately 15 m of the nest. The microphone was oriented directly upwards for all recordings. Recording inputs were maintained constant at a sampling rate of 48 kc s^−1^ and 16-bit resolution, 100% recording volume and 90° microphone configuration.

We uploaded recordings of ambient noise in Raven Pro 1.5, using a Hann window function with a fast Fourier transformation length of 512 samples, 3 dB bandwidth set at 135 Hz and overlap of 50%. The average power of ambient noise was measured by selecting all frequencies across the entire 30 s ambient noise recording, as well as two smaller frequency bands: 0–3000 Hz (the frequency range in which infrastructure noise is loudest) and 3000–12 000 Hz (which includes the frequency range that overlaps with Savannah sparrow alarm calls). The average power was measured from power spectra. We calibrated noise levels by playing a recording of white noise of known sound pressure level, as determined using a Bruel and Kjaer 2250 SPL meter and frequency analyser (C-weighting) 50 cm from the microphone, and digitizing the recording in Raven Pro 1.5. We used the difference between the Raven-reported sound pressure level and the actual sound pressure level of white noise to calculate the actual sound pressure level of ambient noise.

## 3. Statistical analysis

### 3.1. Infrastructure

We used generalized linear mixed-effects models to assess the effects of infrastructure treatment on feeding latency following conspecific alarm calls and western meadowlark songs, and on baseline feeding visits when no stimuli were played. We treated playback type (conspecific alarm call, western meadowlark song, baseline), infrastructure treatment (gas compressor station, generator-powered screw pump, grid-powered screw pump, control) and their interactions as fixed effects, and nest ID as a random effect. To determine whether effects varied with distance from each infrastructure treatment, we modelled the effects of playback type, infrastructure treatment, distance from infrastructure and their interactions on feeding latency. Where we found a significant effect of treatment or distance on feeding latency during baseline feeding visits, we re-ran models on each playback treatment separately, so that we could identify the effects of noise on each playback type independently as well as relative to baseline behaviour. To determine whether infrastructure treatment and distance from each type of infrastructure altered relative responsiveness to conspecific alarm calls and western meadowlark songs, we compared feeding latency following conspecific alarm calls with feeding latency following western meadowlark songs.

### 3.2. Ambient noise

We used generalized linear mixed-effects models to assess the effects of broadband (0–24 000 Hz), low-frequency (0–3000 Hz) and high-frequency (3000–12 000 Hz) noise on feeding latency following the playback of conspecific alarm calls and western meadowlark songs, and on baseline feeding visits when no stimuli were played. We treated playback type, sound pressure level and their interactions as fixed effects, and nest ID as a random effect. Where we found a significant effect of noise on feeding latency during baseline feeding visits, we ran models on each playback treatment separately, as above. To determine whether noise altered relative responsiveness to conspecific alarm calls and western meadowlark songs, we repeated these analyses, comparing feeding latency following conspecific alarm call playbacks with feeding latency following western meadowlark song playbacks.

All analyses were completed using the SAS 9.1 statistical software. Generalized linear mixed-effects models were computed using the Glimmix Procedure, and model means and standard errors of categorical variables were calculated using the lsmeans statement. We determined the distribution of response variable residuals using diagnostic graphs and the deviance/*df* ratio. The variable ‘feeding latency' fitted a negative binomial distribution for all models. Effects were considered significant at *α* = 0.05.

## 4. Results

### 4.1. Response to alarm calls under natural conditions

**Figure 3.**
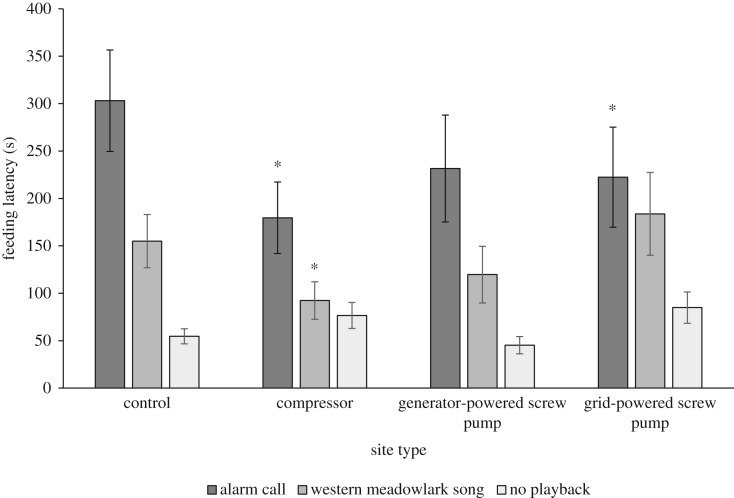
Average feeding latency (± s.e.) of Savannah sparrows (*Passerculus sandwichensis*) in southern Alberta during May–July 2013 and 2014, following the playback of conspecific alarm calls and western meadowlark songs, and on baseline feeding visits when no stimuli were played (no playback), at the control sites (*n *= 21), compressor station sites (*n *= 15), grid-powered screw pump sites (*n *= 12) and generator-powered screw pump sites (*n *= 11). Asterisks indicate feeding latencies for each playback type that differ significantly from those on control sites.

On control sites, Savannah sparrows responded to both conspecific alarm calls and western meadowlark songs by delaying feeding visits. On average, Savannah sparrows took five times longer to return to the nest following the onset of conspecific alarm call playbacks than on baseline feeding visits when no stimuli were played (*β *= 1.7138, s.e.= 0.2015*,* d.f.* *= 153, *t *= 8.51, *p *< 0.0001), while they took three times longer to return to the nest following the onset of western meadowlark song playbacks (*β *= 1.0431, s.e.* *= 0.2067, d.f.* *= 153, *t *= 5.05, *p *< 0.0001) than on baseline feeding visits (figure 3).

While Savannah sparrows responded to both sets of stimuli by delaying feeding visits, this delay was more dramatic when conspecific alarm calls were played. On average, Savannah sparrows took twice as long to return to the nest following the playback of conspecific alarm calls as they did following the playback of western meadowlark songs (*β *= 0.687, s.e.* *= 0.2404, d.f.* *= 55, *t *= 2.86, *p *= 0.006; figure 3).

### 4.2. Effects of oil and gas infrastructure

Savannah sparrows returned to the nest faster at compressor station sites following the playback of both conspecific alarm calls (*β *= −0.8612, s.e.* *= 0.3175, d.f.* *= 153, *t *= −2.71, *p *= 0.0074) and western meadowlark songs (*β *= −0.8561, s.e.* *= 0.3273, d.f.* *= 153, *t *= −2.62, *p *= 0.0098), relative to baseline feeding visits, than they did on control sites. At grid-powered screw pump sites, Savannah sparrows also returned to the nest faster following conspecific alarm calls (*β *= −0.7504, s.e.* *= 0.3396, d.f.* *= 153, *t *= −2.21, *p *= 0.0286) relative to baseline feeding visits; however, this effect may have been driven in part by a trend in increased baseline feeding latency at these sites (*β *= 0.441, s.e.* *= 0.2436, d.f.* *= 153, *t *= 1.81, *p *= 0.0722). There was no difference in feeding latency following either playback type between generator-powered screw pump sites and control sites (*p > *0.4351). There were no significant effects of infrastructure treatment on baseline feeding latency (*p > *0.0722) (figure 3).

While Savannah sparrows were less responsive to both sets of stimuli at compressor station sites, infrastructure treatment did not affect the qualitative responsiveness to stimuli: Savannah sparrows continued to take longer to return to the nest following the playback of conspecific alarm calls than they did following the playback of western meadowlark songs, at all infrastructure treatments (*p *> 0.2007) (figure 3).

**Figure 4.**
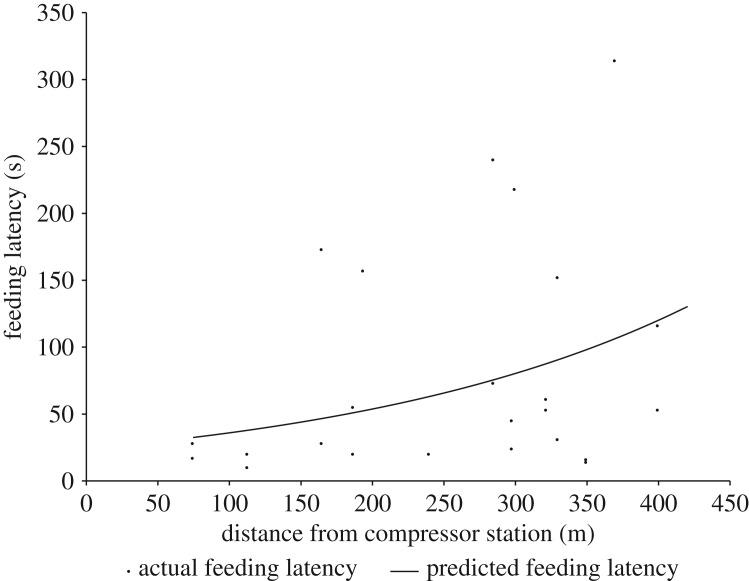
The effects of distance from compressor stations on feeding latency in the Savannah sparrow (*Passerculus sandwichensis*) in southern Alberta during May–July 2013 and 2014 during baseline feeding visits when no stimuli were played. *n *= 15 nests at compressor stations, eight of which had two observations of baseline feeding latency, while seven had only one observation.

Within compressor station sites, Savannah sparrows took less time to approach the nest on baseline feeding visits closer to the infrastructure than they did at the periphery of compressor station sites (*β *= 0.00582, s.e.* *= 0.00228, d.f.* *= 144, *t *= 2.55, *p *= 0.0118; figure 4). There was no effect of distance from infrastructure on feeding latency following either western meadowlark songs or conspecific alarm playbacks relative to baseline feeding visits (*p > *0.219). There was no effect of distance from generator- or grid-powered screw pump sites on feeding latency on baseline feeding visits or following either playback type (*p > *0.3276). There was no effect of distance from infrastructure on the relative difference in feeding latency between conspecific alarm calls and western meadowlark songs (*p *= 0.0852).

### 4.3. Effects of ambient noise

Owing to weather and logistical constraints, ambient noise recordings were only available for 41 out of the 60 nests at which experiments occurred. Broadband noise ranged from 41 to 73 dB(C) (mean = 59 dB(C)), and was louder in the low-frequency range (50–82 dB(C), mean = 68 dB(C)) than in the high-frequency range (21–48 dB(C), mean = 30 dB(C)).

**Figure 5.**
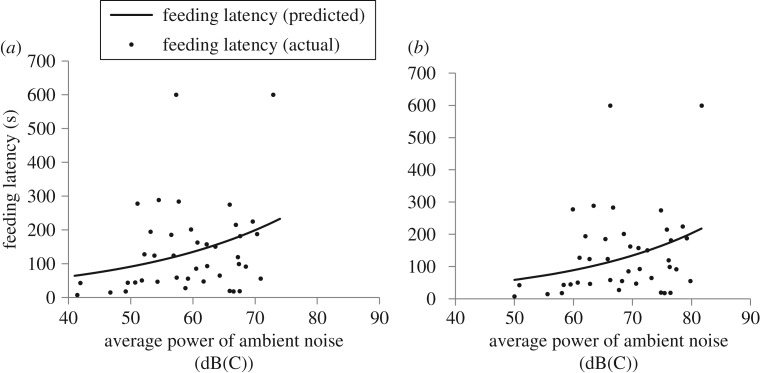
The effects of (*a*) broadband (0–24 000 Hz) and (*b*) low-frequency (0–3000 Hz) ambient noise on Savannah sparrow (*Passerculus sandwichensis*) feeding latency following the playback of western meadowlark songs in southern Alberta during May–July 2013 and 2014; *n* = 41 nests.

Savannah sparrows took longer to approach the nest following western meadowlark song playbacks, relative to baseline feeding visits, at increasing amplitudes of broadband (*β *= 0.04149, s.e.* *= 0.02133, d.f.* *= 99, *t *= 1.95, *p *= 0.0546) and low-frequency noise (*β *= 0.04135, s.e.* *= 0.02127, d.f.* *= 100, *t *= 1.94, *p *= 0.0547), but not high-frequency noise (*p *= 0.2179) (figure 5). There was no effect of noise level on feeding latency on baseline feeding visits or following the playback of conspecific alarm calls (*p > *0.7327). There were no effects of ambient noise on the relative difference in feeding latency between conspecific alarm calls and western meadowlark songs (*p > *0.1022).

## 5. Discussion

Overall, these findings support the hypothesis that Savannah sparrows respond to conspecific alarm calls by delaying feeding visits, and that this response is impaired by noise-producing infrastructure. Under natural conditions Savannah sparrows took longer to approach their nests following the playback of conspecific alarm calls than they did following control western meadowlark song playbacks and on baseline feeding visits when no stimuli were played, but at compressor station sites Savannah sparrows approached their nests sooner than they did on control sites. This suggests that very loud infrastructure noise may prevent Savannah sparrows from responding appropriately to alarm calls, although moderate noise from oil wells may be insufficient to interfere with anti-predator communication. At louder ambient background noise levels, Savannah sparrows also increased feeding latency following playbacks of western meadowlark songs, suggesting that noise may interfere with the ability of Savannah sparrows to discriminate non-adaptive sounds in their environment from biologically relevant acoustic signals such as alarm calls [27,28].

In the absence of anthropogenic ambient noise, Savannah sparrows responded to conspecific alarm calls by delaying feeding visits. This may be an effective anti-predator strategy for grassland breeding songbirds that build their nests directly on the ground [32], as their nests are especially vulnerable to predators. Indeed, nest predation is the leading cause for nest failure in grassland songbirds [40], and thus these species must rely on well-camouflaged nests and cryptic behaviours to prevent their nests from being detected. Like some other bird species (e.g. the Siberian jay (*Perisoreus infaustus*) [41]), Savannah sparrows may be able to prevent their nests from being depredated by temporarily avoiding the area when a predator is in the vicinity to conceal their nest location, and thus awareness of predation risk is critical to the protection of the nest. Because many grassland nest predators, such as rodents and snakes [42], are inconspicuous, parental alarm calls may function as a crucial warning system for mated pairs to signal to each other if a predator has been spotted. A similar response to alarm calls has been documented in female red-winged blackbirds [4,43], which delay feeding visits in response to male alarm calls. However, this is the first study that we are aware of to document this behaviour in Savannah sparrows.

Savannah sparrows also responded to the playback of western meadowlark songs by delaying feeding visits relative to baseline behaviour; however, they paused for less than half of the time that they did following the playback of conspecific alarm calls. While any unexpected noise in the vicinity of their nests may be an indication of danger, birds must balance the pressure to protect their nests from potential predators with the need to provide sufficient food to their offspring [44]. Owing to the extreme vulnerability of their nests [32,40,42], Savannah sparrows may benefit from pausing to assess whether or not a danger is present after unexpectedly hearing any foreign noise close to their nest, but they also benefit from returning to feeding sooner than when they hear alarm calls, which are a reliable indicator of danger.

Of all infrastructure treatments, compressor stations had the greatest effect on anti-predator behaviour during playback trials. At compressor station sites Savannah sparrows approached their nests sooner following both conspecific alarm calls and western meadowlark song playbacks, relative to baseline feeding visits, than they did on control sites. Within compressor station sites, they also reduced feeding latency on baseline feeding visits close to the infrastructure, suggesting less caution in general during provisioning. Taken together, these findings suggest that Savannah sparrows may have been less vigilant when provisioning nestlings in the vicinity of compressor stations.

There are multiple mechanisms that may lead to reduced vigilance in the vicinity of noisy infrastructure. Acoustic masking may prevent Savannah sparrows from hearing playbacks, and therefore they may fail to display the appropriate response of delaying feeding visits [25]. It is also possible that noise may distract Savannah sparrows by adding an additional stimulus to focus on, which may hinder their ability to perform anti-predator behaviour with the same proficiency as they would under quiet conditions [29]. Finally, differences in anti-predator behaviour between the compressor station and the control sites could result from differences in predator abundances between the compressor station and the control sites [21]. We evaluate the evidence for each of these hypotheses below.

While many previous studies have attributed impacts of anthropogenic noise on communication to acoustic masking [14,18], there are some inconsistencies between the predicted effects of masking and the findings of this study. The potential for acoustic masking increases with the amplitude of ambient noise [25,33] and the degree to which ambient noise overlaps in frequency with acoustic signals [45]. However, we found no reduction in feeding latency with increases in ambient noise, including within the 3000–12 000 Hz range, which overlaps in frequency with Savannah sparrow alarm calls. Furthermore, acoustic masking cannot explain why Savannah sparrows reduced feeding latency close to compressor stations during baseline feeding visits when no stimuli were played.

Lower feeding latency following playbacks at compressor station sites may be better explained by the distracting effects of anthropogenic noise than by masking. According to the distracted prey hypothesis, anthropogenic noise can reduce responsiveness to signals by providing an additional stimulus that animals must focus on [29]. While it can be difficult to distinguish between masking and distraction, there are some differences in how these two mechanisms act on acoustic signals. While acoustic masking increases with the amplitude of ambient noise [25,33] and the degree to which ambient noise overlaps in frequency with acoustic signals [45], distraction is independent of both these factors [31,46]. Unlike the other infrastructure treatments, compressor stations can be heard by the human ear up to a kilometre away (B. Antze 2014, personal observation), but the noise they produce may not contribute substantially to the overall sound pressure level of noise throughout the compressor station sites when other natural noise sources are present, such as wind, insects and other birds. Distraction may also explain why Savannah sparrows reduced feeding latency during baseline feeding visits closer to compressor stations, as noise may distract Savannah sparrows from performing normal pre-provisioning anti-predator scanning [13].

Physical structures associated with energy infrastructure may also contribute to their impact on anti-predator communication and behaviour, and this may further explain why compressor stations had a greater impact than screw pumps. Unlike screw pumps, compressor stations include multiple large vertical structures, such as turbines, sheds and other metallic buildings. The reflective surfaces associated with these structures can cause reverberations that mask or blend call features, degrading the quality of acoustic signals, while simultaneously projecting or amplifying the noise produced by compressor stations, through flutter-echo effects [24]. Furthermore, as distraction can be multimodal [29], visual disturbance from the turbines may further limit the attentional abilities of Savannah sparrows, by providing an additional dynamic visual stimulus to their environment.

An alternative explanation for the differences in anti-predator behaviour between compressor station sites and control sites is that Savannah sparrows may be responding to differences in predator abundances among sites, rather than noise. While predator abundances were not directly measured in this study, it has been shown elsewhere that some nest predators may avoid noise-producing infrastructure [21], and therefore the less vigilant behaviour displayed here may be appropriate, as this allows Savannah sparrows to focus more time on provisioning nestlings. This could explain the decrease in baseline (no-playback) feeding latency closer to compressor stations, as Savannah sparrows may require less time to evaluate risk before provisioning nestlings. However, it seems unlikely that predator abundances at breeding sites would affect responses to alarm calls, as alarm calls should be perceived as a reliable signal that a threat is present, regardless of the relative abundance of predators at a particular site. Furthermore, we have shown elsewhere that 39% of grassland songbird nests in the study area were depredated over the study period [42], with no significant differences in nest success between the compressor station and the control sites, indicating that Savannah sparrows should be under especially strong selective pressure to respond to these signals if they are to protect their nests.

At grid-powered screw pump sites Savannah sparrows also had lower feeding latency following alarm calls relative to baseline feeding visits; however, this appeared to be driven primarily by a trend in increased feeding latency during baseline feeding visits. It is unlikely that this trend was caused by infrastructure noise, as grid-powered screw pumps were quieter than generator-powered screw pumps, which had no effect on anti-predator behaviour. Savannah sparrows may be more vigilant during baseline feeding visits at grid-powered screw pump sites because of higher predator abundances; a study using the same sites and nests found that predation levels of Savannah sparrow nests were higher at grid-powered screw pump sites than on control sites or generator-powered well sites, perhaps because avian predators use power distribution lines as perch sites [42].

Surprisingly, increases in ambient noise were not linked to a decrease in feeding latency following either playback type; on the contrary, Savannah sparrows increased feeding latency following western meadowlark song playbacks at higher amplitudes of ambient noise. Heightened responsiveness to inappropriate stimuli, such as western meadowlark songs, can be characterized as ‘false alarms’ [28], which occur when animals are not able to clearly discriminate acoustic information [25], and hence increase their response threshold to all stimuli to ensure they continue to respond properly to biologically appropriate stimuli such as alarm calls. If ambient noise prevents Savannah sparrows from hearing all elements within western meadowlark songs, it may take longer for them to ascertain that these songs are not a reliable indicator of danger. However, false alarms come at a cost: if Savannah sparrows waste too much time pausing to assess threats that are not real, they lose valuable time that could be spent provisioning nestlings [44].

This elevated response to western meadowlark song playbacks with noise is better explained by acoustic masking than distraction. As predicted by masking [25,33], post-playback feeding latency increased with the amplitude of ambient noise, and appeared to be driven primarily by low-frequency noise (0–3000 Hz), which overlaps with the range occupied by western meadowlark songs (approx. 1500–4700 Hz; figure 2). Feeding latency following western meadowlark song playbacks did not increase at noisier infrastructure, suggesting that this effect may be driven primarily by natural noise sources in the environment, such as wind, insects and other birds. Given that these factors have been present throughout the evolutionary history and individual lives of Savannah sparrows, it seems unlikely that these natural noise sources would significantly affect their attentional abilities, and thus these effects are less likely to be caused by distraction.

While this study demonstrated that Savannah sparrows are less responsive to alarm calls in the presence of noise-producing infrastructure, many birds are able to alter the structure of songs [33,34,47,48] and alarm calls [37,38] in the presence of noise, in order to overcome interference. Thus, if Savannah sparrows alter the structure of alarm calls close to noisy infrastructure such as compressor stations, noise may present less of a barrier than suggested by this study. However, the degree to which altering vocalizations may help Savannah sparrows to improve responsiveness to alarm calls depends on which mechanisms drive the effects of noisy infrastructure on anti-predator behaviour. If reduced responsiveness to alarm calls at compressor station sites is driven by acoustic masking [25], altering alarm calls may improve signal transmission. However, if effects are driven by distracting effects of infrastructure noise [29], altering alarm calls may have little effect on the ability of Savannah sparrows to display appropriate anti-predator behaviour at these sites. Given that the impacts of compressor stations on alarm communication seem to be more consistent with distraction, this suggests that, even if Savannah sparrows do alter the structure of alarm calls at these sites, they may not be able to fully overcome the negative effects of noise on anti-predator behaviour. Further work could directly address this question by determining whether Savannah sparrows are more responsive to altered calls than unaltered calls in the presence of noise-producing infrastructure.

Taken together, our results suggest that the effects of infrastructure on anti-predator behaviour may be distinct from the effects of ambient noise levels. Importantly, this suggests that noise reduction mechanisms alone may not be an effective means of reducing the impacts of oil and gas extraction structure on songbirds. As we observed the greatest effects from infrastructure with the largest acoustic and structural footprint, decreasing the spatial extent, visual impact and acoustic disturbance of infrastructure may all be necessary to reduce distraction of Savannah sparrows from their reproductive tasks. The effects of distraction and masking are not mutually exclusive [29], and thus it is not surprising that both seem to influence avian behaviour in this system. Our observation that Savannah sparrows are less responsive to anti-predator signals in the vicinity of natural gas compressor stations is of conservation concern, and adds to a growing body of evidence that noisy anthropogenic structures have the potential to negatively affect birds by interfering with acoustic communication [16–18,49].

## Supplementary Material

Supporting Data

## Supplementary Material

SAS code

## Data accessibility

The dataset supporting this article have been uploaded as the electronic supplementary material.
