# Supplementary material for: Noisy anthropogenic infrastructure interferes with alarm responses in Savannah sparrows (Passerculus sandwichensis)
Source: R Soc Open Sci. 2018 May 16;5(5):172168. doi: 10.1098/rsos.172168 (PMC5990837; doi:10.1098/rsos.172168)
Supplement: Supporting Data [file rsos172168supp1.docx]

**SAS code:**

Site type

**proc glimmix** data=work.data plots=meanplot method=laplace;
class Nest_number playback_type site_type;
model feeding_latency = playback_type site_type playback_type*site_type/ solution dist=negbin;
random intercept / subject=Nest_number type=vc;*cs or ar(1);
output out=residual_out pred = predicted_values resid =residuals UCL=upper lcl=lower ;
lsmeans playback_type*site_type / ILINK;
**run;**

Distance from infrastructure

**proc** **glimmix** data=work.data method=laplace;

class Nest_number playback_type site_type;

model feeding_latency = playback_type site_type distance_from_infrastructure_cen playback_type*site_type playback_type*site_type*distance_from_infrastructure_cen/ solution dist=negbin;

random intercept / subject=Nest_number type=vc;*cs or ar(1);

output out=residual_out pred = predicted_values resid =residuals UCL=upper lcl=lower;

**run**;

| \| **PROC** **GENMOD** data=alarm_only; \| \| --- \| \| class site_type; \| \| model feeding_latency = site_type distance_from_infrastructure_cen site_type*distance_from_infrastructure_cen/ dist=negbin; \| \| output out=residual_out pred = predicted_values Resraw =residuals Upper=UCL Lower=lcl; \| \| **run**; \| \| **PROC** **GENMOD** data=weme_only; \| \| class site_type; \| \| model feeding_latency = site_type distance_from_infrastructure_cen site_type*distance_from_infrastructure_cen/ dist=negbin; \| \| output out=residual_out pred = predicted_values Resraw =residuals Upper=UCL Lower=lcl; \| \| **run**; \| |
| --- | --- | --- | --- | --- | --- | --- | --- | --- | --- | --- |

Ambient noise

| **proc** **glimmix** data=work.data method=laplace; |
| --- |
| class Nest_number playback_type site_type; |
| model feeding_latency = playback_type ambient_noise_0_24000Hz playback_type*ambient_noise_0_24000Hz/ solution dist=negbin; |
| random intercept / subject=Nest_number type=vc;*cs or ar(1); |
| output out=residual_out pred = predicted_values resid =residuals UCL=upper lcl=lower; |
| **run**; |

| **proc** **glimmix** data=work.data method=laplace; |
| --- |
| class Nest_number playback_type site_type; |
| **model feeding_latency = playback_type ambient_noise_0_3000Hz playback_type*ambient_noise_0_3000Hz/ solution dist=negbin;** |
| **random intercept / subject=Nest_number type=vc;*cs or ar(1);** |
| **output out=residual_out pred = predicted_values resid =residuals UCL=upper lcl=lower;** |

| **run;**  **proc glimmix data=work.data method=laplace;**  **class Nest_number playback_type site_type;**  **model feeding_latency = playback_type ambient_noise_3000_12000Hz playback_type*ambient_noise_3000_12000Hz/ solution dist=negbin;**  **random intercept / subject=Nest_number type=vc;*cs or ar(1);**  **output out=residual_out pred = predicted_values resid =residuals UCL=upper lcl=lower;**  **run;** |
| --- |
